# Beyond the target: implications of Wnt pathway inhibitors on bone health

**DOI:** 10.1007/s00109-026-02697-3

**Published:** 2026-07-04

**Authors:** Clement Nachef, Arnaud Vanjak, Eric Haÿ, Martine Cohen-Solal, Thomas Funck-Brentano

**Affiliations:** 1 Rheumatology Department, Lariboisiere Hospital, Paris, France; 2 Université de Paris, Paris, France; 3 INSERM 1132 Unit, 2 Rue Ambroise Pare, 75010 Paris, France

**Keywords:** Wnt signaling pathway, Bone health, Fracture

## Abstract

Wnt signaling inhibitors are under investigation as potential therapies for conditions characterized by upregulated Wnt signaling, such as cancers, hematological disorders, and organ fibrosis. However, because the Wnt pathway is essential for bone homeostasis, its inhibition can adversely affect skeletal health. This review focuses on the bone-related off-target effects of Wnt inhibitors currently in clinical development, specifically those evaluated in Phase I and II trials with available published data. We found that inhibitors targeting upstream components of the pathway—such as Wnt ligands or receptors (e.g., porcupine inhibitors, Ipafricept, or Vantictumab)—frequently lead to bone-related adverse effects, including fractures and early trial termination. Co-administration of bisphosphonates may help mitigate these effects. In contrast, downstream inhibitors (e.g., PRI-724, niclosamide) have not been linked to bone toxicity, although this may reflect either underreporting or a genuinely lower skeletal impact. Further preclinical studies are warranted to better understand these differential effects. A thorough understanding of bone-specific risks is critical as Wnt signaling inhibitors continue to advance in clinical development.

## Introduction

The Wnt signaling pathway is an ubiquitous signaling pathway involved in numerous cellular behaviors such as proliferation, migration, or differentiation. Various components participate in its regulation: ligands, receptors, cytoplasmic, and nuclear components. Its dysregulation can lead to severe consequences, including organ dysfunction and the development of cancers. Mutations in molecular partners in Wnt are found in several genetic diseases.

The discovery of the Wnt pathway occurred independently in Drosophila and mice around the 1980s. In Drosophila, the "wingless" gene was identified in wingless flies as a gene involved in embryo polarity [1]. In mice, the "int-1" gene was discovered as a proto-oncogene involved in mammary tumors [2]. It was later identified that these two genes coded for homologous proteins, and the fusion of "wingless" and "int-1" gave rise to "Wnt."

Wnt inhibitors are being investigated for their potential therapeutic benefits in a variety of diseases where the Wnt signaling pathway is upregulated. Notable applications include oncology, where their use is particularly prominent, fibrotic disorders such as hepatic fibrosis, and other conditions like endometriosis. Because tightly regulated Wnt signaling is essential for cellular differentiation and the regeneration of various tissues—particularly those with high cellular turnover such as hematopoietic and gastrointestinal tissues—its inhibition poses significant risks of adverse effects. In clinical studies, the most frequently reported side effects associated with Wnt signaling inhibition include gastrointestinal disturbances, alopecia, immunosuppression, fatigue, vitiligo, anemia, neutropenia, thrombocytopenia, bone fractures, and neurodegeneration [3].

The aim of this review is to specifically explore the bone-related off-target effects of Wnt signaling inhibitors. Given the rapidly growing number of Wnt inhibitors in development, we focused on those that have advanced to clinical stages. A comprehensive literature search was conducted to identify relevant studies published between 2010 and June 2025. PubMed and ClinicalTrials.gov were systematically searched without restriction on clinical indication. Only studies reporting clinical data were included; preclinical studies, restricted to *in vitro* and animal experiments, were excluded. Titles and abstracts were screened for relevance, and full texts of potentially eligible studies were reviewed to determine final inclusion.

## Wnt signaling pathways

### Canonical Wnt signaling pathway

The principal actor in the canonical Wnt pathway is the β-catenin protein, encoded by the *Ctnnb1* gene. This protein was initially identified in Drosophila under the name Armadillo as a protein involved in the interaction between cadherin and cytoskeleton [4]. Another source of β-catenin was later identified in the cytoplasm and nucleus, and associated with the canonical Wnt signaling pathway [5].

#### Basal situation (Fig. 1)

In the absence of Wnt ligand binding to LRP and Frizzled receptors, cytoplasmic β-catenin is recruited by a degradation complex composed of Axin2, Adenomatous Polyposis Coli (APC), and GSK3β proteins [6]. Thus, β-catenin is phosphorylated at specific segments by GSK3β [7, 8] and CK1 proteins [8, 9]. It is then recognized by proteins such as the F-box protein β-TrCP [10], leading to its ubiquitination and proteasomal degradation [11].

**Fig. 1:**
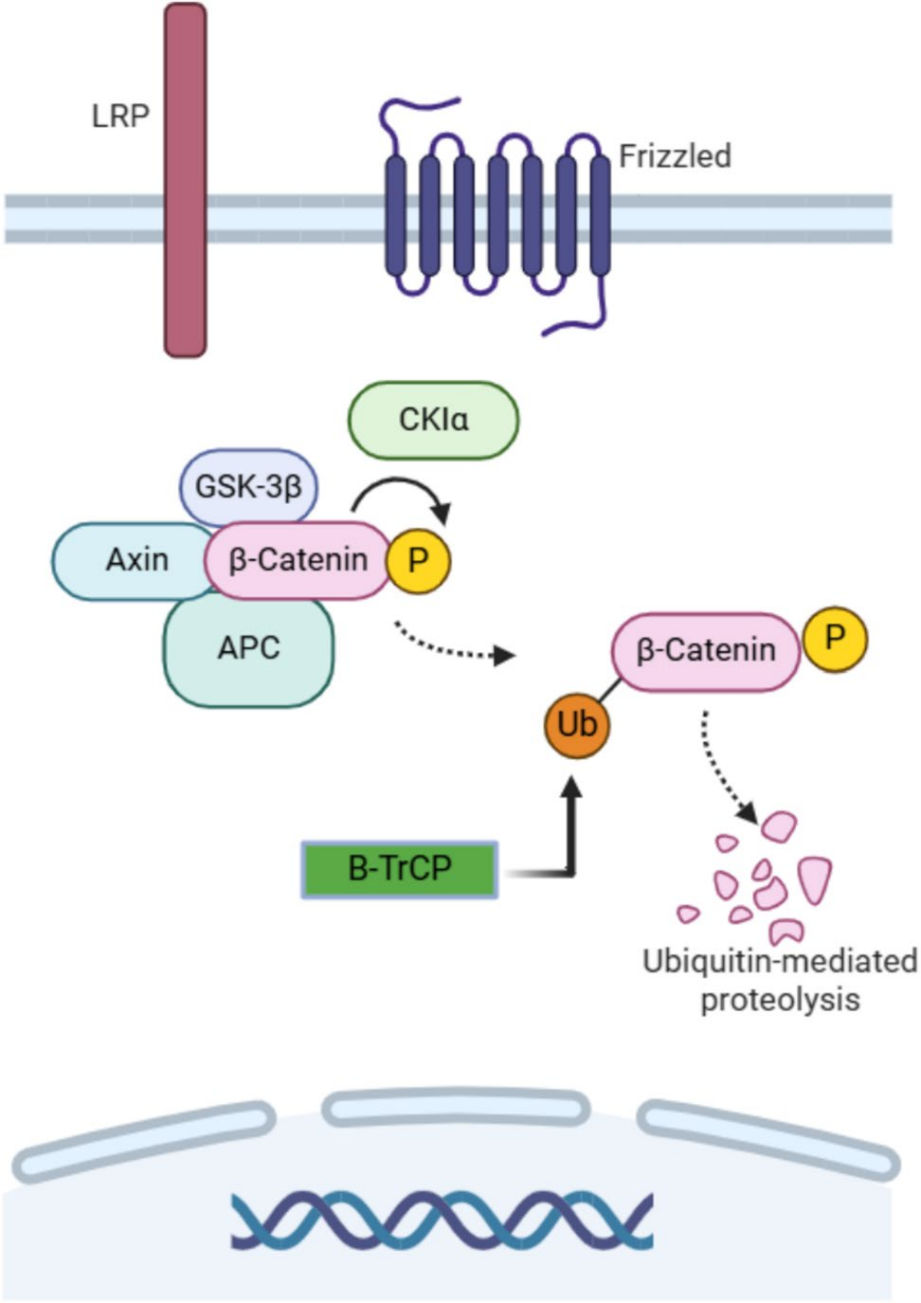
Basal situation of Wnt signaling pathway (*Biorender*). In the absence of Wnt ligand binding to LRP and Frizzled receptors, cytoplasmic β-catenin is recruited by a degradation complex composed of Axin2, Adenomatous Polyposis Coli (APC), and GSK3β protein. Thus, β-catenin is phosphorylated at specific segments by GSK3β [7, 8] and CK1 proteins. It is then recognized by proteins such as the F-box protein β-TrCP leading to its ubiquitination and proteasomal degradation

#### Activation of the Wnt pathway (Fig. 2)

Prior to receptor binding, Wnt ligands undergo a post-translational modification known as palmitoylation, essential for the release outside the cell and the interaction with the receptors at the membrane. This function is performed by the enzyme Porcupine in the endoplasmic reticulum [12]. Another protein, Wntless, plays a role in Wnt ligand secretion, homing from the Golgi to the cell membrane [12].

**Fig. 2:**
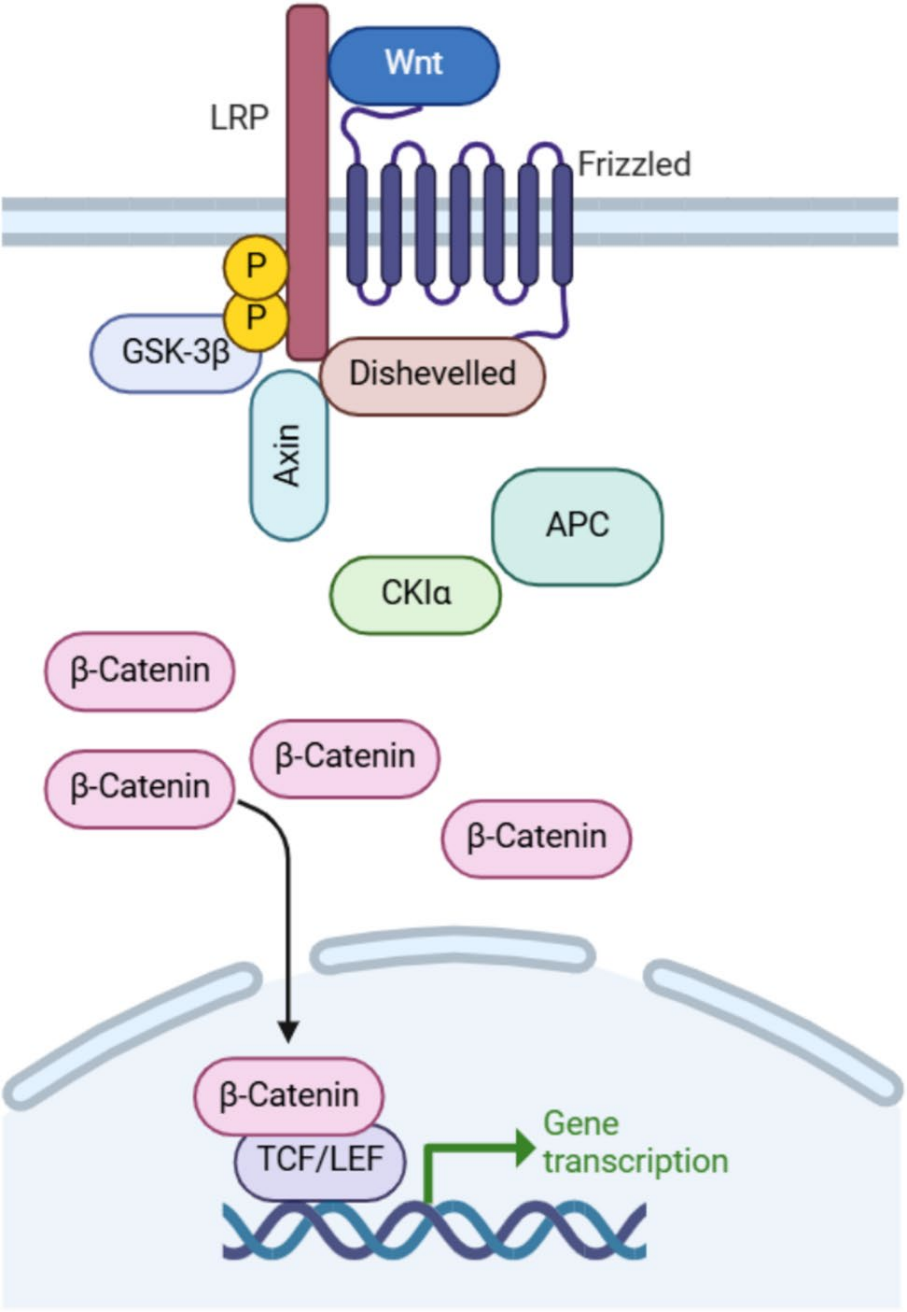
Activation of canonical Wnt signaling pathway (*Biorender*). Upon Wnt ligand binding, LRP and Frizzled receptors dimerize, inducing a conformational change with phosphorylation of the cytoplasmic parts of LRPs. GSK3β recognizes these phosphorylation sites, resulting in the inhibition of the degradation complex. Another critical event is the binding of Dishevelled to Frizzled, forming a platform for interaction between the cytoplasmic parts of LRPs and Axin. Consequently, the members of the β-catenin degradation complex are sequestered at the plasma membrane, allowing non-phosphorylated β-catenin to accumulate in the cytoplasm. Accumulated β-catenin translocates to the nucleus, where it acts as a co-transcription factor with T-cell factor/lymphoid enhancer-binding factor (TCF/LEF) or CREB-binding protein (CBP)

Upon ligand binding, LRP and Fzd receptors dimerize [13], inducing a conformational change with phosphorylation of the cytoplasmic parts of LRPs. GSK3β recognizes these phosphorylation sites and binds to them [14], resulting in the inhibition of the degradation complex. Another critical event is the binding of Dishevelled (DVL) to Frizzled (Fzd) [15, 16], forming a platform for interaction between the cytoplasmic parts of LRPs and Axin [17]. Consequently, the members of the β-catenin degradation complex are sequestered at the plasma membrane [18], allowing non-phosphorylated β-catenin to accumulate in the cytoplasm. Accumulated β-catenin translocates to the nucleus, where it acts as a co-transcription factor with T-cell factor/lymphoid enhancer-binding factor (TCF/LEF) [19] or CREB-binding protein (CBP) [20]. Target genes include *Axin2* [21], *DKK1* [22], *CCN1/Cyr61* [23], *RANKL* [24], or *Runx2* [25].

#### Inactivation of the Wnt pathway

The Wnt pathway can be inhibited by blocking Wnt ligands. Proteins such as Sfrp (Secreted Frizzled-Related Proteins), numbered from 1 to 5, inhibit Wnt ligands extracellularly before their interaction with membrane receptors [26]. At the membrane level, Sclerostin (SOST) inhibits LRP5 and LRP6 receptors, thus affecting the canonical Wnt pathway [27, 28]. The Dickkopf (DKK) family, consisting of four members (DKK1 to 4) [29], inhibits the Wnt pathway by causing LRP internalization, preventing Wnt ligand binding [30].

### Non-canonical Wnt signaling pathway

In the absence of LRP5/6 receptors, Wnt ligands can also bind to Frizzled or ROR/RYK receptors, activating the Wnt pathway independently of β-catenin, known as the non-canonical Wnt pathway. The two most studied pathways are the Wnt-Planar Cell Polarity (PCP) pathway and the Wnt-Ca2 + pathway [31].

#### Wnt-PCP pathway

The Wnt-PCP pathway was discovered in the establishment of cell polarity, initially in Drosophila. Upon Wnt ligand binding to Fzd, DVL is recruited and transmits a signal via G proteins (RAC, RhoA), activating c-Jun N-terminal kinase (JNK) phosphorylation, leading to cytoskeletal modifications and cell movement [32]. Other receptors, such as ROR1 or ROR2, also participate in this pathway [33].

#### Wnt-Ca2 + pathway

The Wnt-Ca2 + pathway induces cytoskeletal modifications by regulating intracellular calcium levels. After Wnt ligand binding to Fzd, DVL is recruited and activates phospholipase C (PLC), causing intracellular Ca2 + release from the endoplasmic reticulum [34]. This calcium can activate kinases such as Ca/calmodulin-dependent protein kinase (CAMK2) and protein kinase C (PKC), which are involved in cytoskeletal remodeling and cell migration [35].

### Wnt signaling pathway and osteoblasts

Canonical Wnt signaling promotes the commitment of mesenchymal stem cells toward the osteoblastic lineage while inhibiting alternative differentiation pathways such as adipogenesis. In osteoblasts, this pathway enhances proliferation, maturation, and survival, and stimulates the expression of key transcription factors including *Runx2* and *Osterix*. Genetic and experimental studies have demonstrated that modulation of canonical Wnt signaling critically influences bone mass, highlighting its essential role in bone homeostasis [36, 37].

Non-canonical Wnt signaling encompasses β-catenin–independent pathways, primarily including the planar cell polarity (PCP) and Wnt/Ca^2^⁺ pathways, which regulate osteoblast function through distinct molecular mechanisms. These pathways influence cytoskeletal organization, cell polarity, migration, and intracellular calcium signaling, thereby contributing to the spatial organization and functional activity of osteoblasts during bone formation. Non-canonical Wnt ligands such as Wnt5a and Wnt11 have been shown to modulate osteoblast differentiation and matrix mineralization, often through activation of small GTPases, JNK signaling, or calcium-dependent effectors. Importantly, non-canonical Wnt signaling can interact with or antagonize canonical Wnt activity, providing an additional layer of regulation that fine-tunes osteogenic processes and ensures coordinated bone remodeling and skeletal integrity [38].

### Wnt signaling pathway and osteoclasts

Wnt/β-catenin signaling is a key brake on osteoclast differentiation and resorptive activity, acting both indirectly through osteoblast-lineage cells and, in part, directly on the osteoclast lineage. In basic science models, activation of β-catenin signaling in osteoblasts increases osteoprotegerin (OPG) and shifts the RANKL/OPG balance toward inhibition of osteoclastogenesis, thereby reducing osteoclast number and function [37]. In addition, canonical Wnt signaling can restrain osteoclast differentiation through osteoclast-intrinsic mechanisms (e.g., β-catenin–dependent pathways), highlighting that Wnt control of resorption is not solely mediated by osteoblast-derived OPG. Clinically, these concepts are supported by the effects of sclerostin inhibition: romosozumab enhances Wnt signaling and produces a characteristic “dual effect,” with a rapid rise in bone formation markers (e.g., P1NP) accompanied by a transient suppression of bone resorption markers (e.g., β-CTX/CTX), consistent with reduced osteoclast activity *in vivo* [39].

## Wnt inhibitors in clinical development

### Inhibition of Wnt ligands secretion

Porcupine (PORCN) is an enzyme that palmitoylates Wnt ligands in the endoplasmic reticulum before their secretion into the intercellular space. Inhibitors of PORCN suppress the secretion of all Wnt ligands and thereby inhibit both canonical and non-canonical Wnt signaling pathways. The most extensively studied PORCN inhibitor is LGK-974.

LGK-974 has shown significant bone-related effects. Preclinical studies in wild-type female mice have shown decreased bone formation and increased bone resorption at the trabecular and cortical bone [40]. Additionally, in osteosclerosis mice models, LGK-974 was associated with reduced bone formation [41]. These effects could potentially be mitigated by the concurrent administration of bisphosphonates [42]. Clinical data from Phase IB/II trials in metastatic colorectal cancer also indicated severe bone effects [43]. Specifically, 5 out of 20 patients experienced fractures, and 1 patient developed hypercalcemia, leading to the discontinuation of LGK-974 development due to its toxicity profile.

Another porcupine inhibitor under development is AZD5055. A phase 1 dose-escalation study conducted in healthy volunteers reported no bone fractures [44]. However, further studies are required to assess its potential bone toxicity.

### Extracellular Wnt ligands inhibition

Ipafricept (OMP-54F28) is a ligand inhibitor related to Frizzled-8 (Fzd8). It is a recombinant fusion protein (immunoadhesin) consisting of the extracellular ligand-binding domain of the human Fzd8 receptor, and the human IgG1 Fc fragment [45]. In clinical studies, a Phase I trial reported a doubling of the bone resorption marker CTX, and the occurrence of two fractures (one vertebral and one sacral) [46]. In a subsequent Phase Ib study, metastatic patients were treated with zoledronate or denosumab in cases of elevated CTX levels (7 out of 26 patients). This strategy could effectively prevent fractures during the study [47].

### Inhibitors of Wnt receptors

Vantictumab (OMP-18R5) is a monoclonal antibody that binds to Fzd receptors and inhibits canonical Wnt signaling pathway [48, 49]. Two Phase Ib studies have been conducted. The first, involving patients with metastatic pancreatic cancer, reported incidental fractures in 4 out of 31 patients [50]. The second study, involving patients with HER2-negative breast cancer, found new fractures in 6 out of 48 patients [51]. Due to these observed bone toxicities, the development of Vantictumab was discontinued.

An antagonist of LRP5, BI 905681 has been recently studied in patients with advanced and metastatic solid tumors[52]. No bone toxicity was observed but patients were excluded from this study if they presented an history of osteoporosis, fragility fracture or corticosteroid use.

Niclosamide, originally an anti-helminthic, has recently been discovered to inhibit the Wnt pathway by targeting LRP6 [53]. It may also inhibit Fzd receptors [54] or act intracellularly by blocking TCF/BCl9 [55]. Niclosamide is under development for cancer therapy [56], as well as for other diseases such as endometriosis [57]. *In vitro* studies have shown that niclosamide inhibits both osteoblast differentiation and osteoclastogenesis [58]. However, no bone-related effects have been described in preclinical murine models or in humans [59, 60].

### Wnt inhibitors in the cytoplasm

Pyrvinium, another anti-helminthic, enhances the degradation of β-catenin by the proteasome by increasing the activity of casein kinase 1α (CK1α) [61]. No bone-related effects have been described either *in vitro* or *in vivo*. A recent Phase I study repurposing pyrvinium for the treatment of pancreatic cancer did not report bone toxicity [62].

CWP232291 (CWP291) is another small-molecule inhibitor targeting the Wnt signaling pathway. Its metabolized form, CWP232204 induces endoplasmic reticulum (ER) stress, subsequently activating caspases that lead to a reduction in β-catenin levels. No bone-related effects have been described either *in vitro* or *in vivo*. A recent Phase I study for the treatment of relapsed or refractory acute myeloid leukemia and myelodysplastic syndrome did not report bone toxicity [63].

### Wnt inhibitors in the nucleus

PRI-724 inhibits the Wnt signaling pathway by specifically targeting the CBP-β-catenin complex once β-catenin is intranuclear [64]. It has been developed for cancers involving Wnt pathway dysregulation as well as fibrotic diseases, including liver fibrosis [65], cardiac fibrosis [66], lung fibrosis [67], and also endometriosis [68]. There are no preclinical *in vitro* or *in vivo* data regarding its effects on bone. In clinical studies, a Phase I trial in patients with hepatitis C-induced cirrhosis did not report bone-related toxic events [69]. Ongoing Phase II trials are currently investigating its efficacy in hepatic cirrhosis (NCT03620474) and acute myeloid leukemia (NCT01606579), with results yet to be published.

### Non-canonical Wnt inhibitors

Cirmtuzumab is an anti-ROR antibody developed for hematological malignancies, including lymphoma [70] and chronic lymphocytic leukemia (CLL) [71]. There are no reported bone-related effects either *in vitro* or *in vivo*. A recent Phase I study in CLL patients did not report bone toxicity [72].

**Fig. 3:**
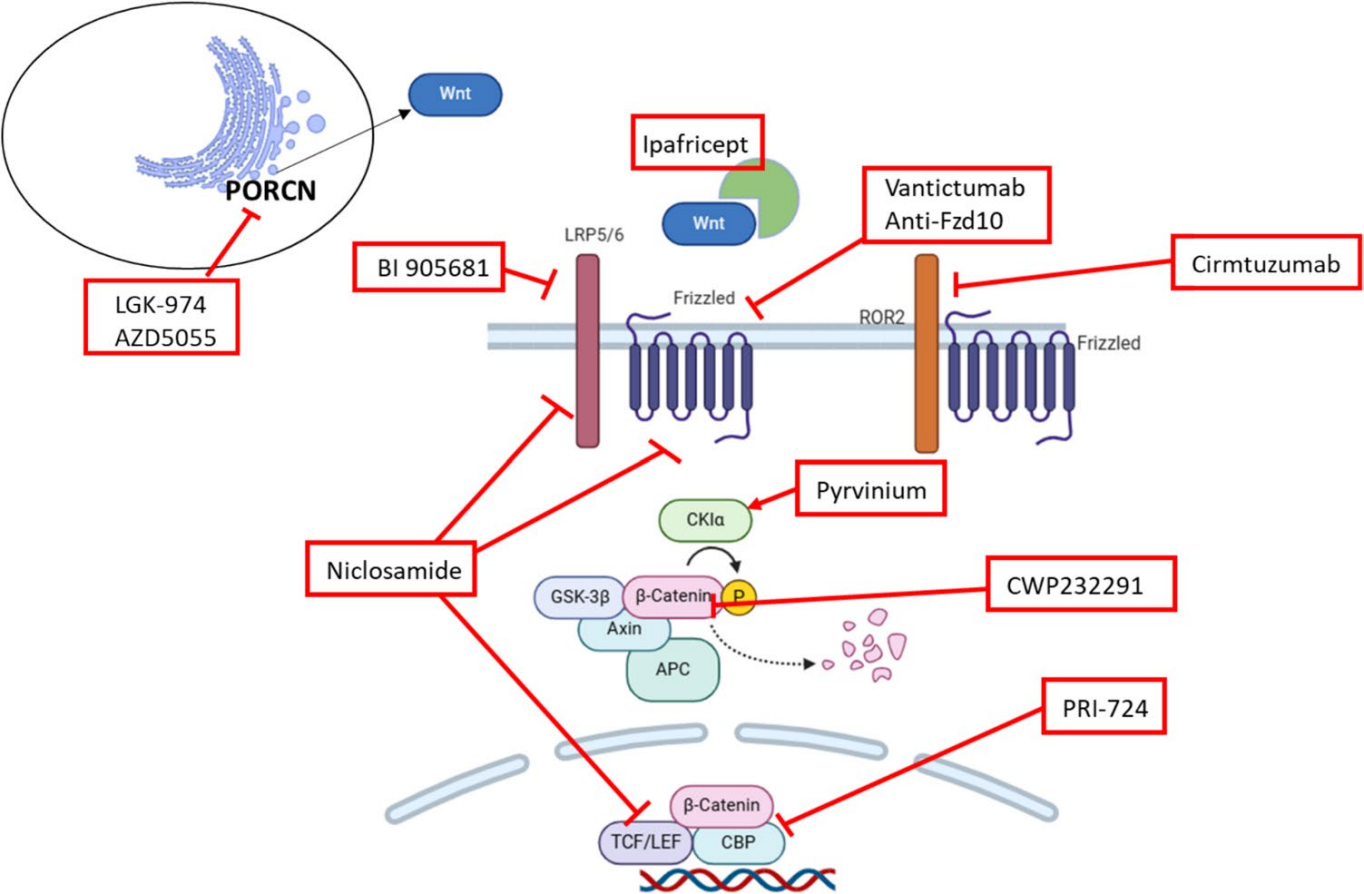
Inhibitors of Wnt signaling pathway *(Biorender)*

In conclusion, Fig. 3 summarizes the Wnt pathway inhibitors discussed in this review with their respective mechanisms of action, while the Table presents their associated effects on bone (Table 1).

**Table 1:** Summary of Wnt signaling pathway inhibitors, including their known mechanisms of action, and reported effects *in vitro, in vivo*, and in clinical studies

Inhibition category	Wnt inhibitor	Preclinical studies	Clinical studies	Bone protection measures
PORCN inhibitors	LGK-974	In mice: decreased bone mass, decreased bone formation and increased bone resorption [40]	In metastatic colorectal cancer: 5 fractures in 20 patients (25%) [75] NCT02278133	-
	AZD5055	-	No fracture reported in healthy volunteers[44] NCT05134727	-
Inhibition of Wnt ligand	Ipafricept	-	In advanced solid tumors: 2 fractures in 26 patients (8%) [46] NCT01608867	Zoledronic acid in patients with elevated CTX [47]
Inhibition of Wnt receptors	Vantictumab	-	In metastatic pancreatic cancer: 4 fractures in 31 patients (13%) [50] In breast cancer: 6 fractures in 48 patients (13%) [51] NCT01973309	-
	LRP5 inhibitor BI 905681	-	No fracture reported [52] NCT04147247	Exclusion of osteoporotic patients
	Niclosamide	In vitro study: decreased osteoblast differentiation and decreased osteoclastogenesis [58]	No fracture reported [59, 60] NCT04399356 NCT04644705	-
Wnt inhibitor in the cytoplasm	Pyrvinium	-	No fracture reported [62] NCT05055323	-
	CWP232291	-	No fracture reported [63] NCT01398462	-
Inhibition of β-catenin	PRI-724	-	No fracture reported [69] NCT02195440	-
Non canonical Wnt inhibitor	Cirmtuzumab	-	No fracture reported [72]	-

Bone protection measures are presented in the right column

## Discussion

Historically, Wnt inhibitors were developed in oncology following the discovery of tumors exhibiting dysregulation of the Wnt signaling pathway. Subsequently, these inhibitors have also been explored for fibrotic diseases, where aberrant Wnt signaling plays a contributory role. However, the Wnt signaling pathway is crucial for bone tissue homeostasis. Variants in genes encoding components of this pathway have been associated with various skeletal consequences, including limb malformations, dental anomalies, and bone fragility [73, 74].

Conversely, therapeutic enhancement of Wnt signaling in bone has led to the development of anabolic agents such as romosozumab, which increases bone mass, reduces bone resorption, and thereby lowers fracture risk in osteoporotic patients [39].

There is clear evidence that Wnt-inhibiting therapies can have detrimental effects on bone health by simultaneously decreasing bone formation and increasing bone resorption. Inhibiting the pathway at the ligand level—such as with porcupine inhibitors, which block all Wnt ligands from both canonical and non-canonical branches—has been associated with significant skeletal toxicity [75]. Similarly, targeting Wnt ligands with agents like Ipafricept [46, 47] or neutralizing several Frizzled (Fzd) receptors using monoclonal antibodies such as vantictumab [50, 51], has resulted in serious bone-related adverse events, including fractures. These toxicities have led to early termination of several Phase II trials. [42, 47].

In contrast, no fractures have been reported in clinical studies involving downstream inhibitors of the Wnt pathway—those acting intracellularly at the level of β-catenin—such as PRI-724, niclosamide, or pyrvinium [59, 60, 62]. This absence may reflect underreporting, or alternatively, a genuinely lower degree of bone toxicity. One hypothesis is that intracellular inhibition elicits compensatory mechanisms that limit skeletal impact. Feedback loops involving intranuclear transcription cofactors may play a role, as evidenced by the regulation of p300 in response to CBP inhibition by PRI-724 [76].

However, this apparent safety profile should be interpreted with caution. Tegavivint, a selective β-catenin/TBL1 inhibitor currently under clinical investigation in hepatocellular carcinoma, has been associated with decreased bone mass in preclinical mouse models [77], suggesting that intracellular targeting of the Wnt pathway may still adversely affect skeletal homeostasis. These findings indicate that the level of pathway inhibition alone may not fully predict bone toxicity, and that compound-specific effects and context-dependent mechanisms are likely involved. Overall, dedicated preclinical *in vitro* and *in vivo* studies remain essential to better characterize the skeletal impact of Wnt pathway inhibitors acting at different levels.

Ultimately, the bone-related adverse effects observed in oncology trials underscore the central role of Wnt signaling in skeletal homeostasis. General measures applicable to drug-induced osteoporosis should be systematically considered in patients exposed to therapies that may impair bone health [78]. Patients with pre-existing bone fragility may be at increased risk of subsequent fragility fractures and should therefore be carefully identified. In clinical practice, patients at high fracture risk should be proactively screened on the basis of prior fracture history, established clinical risk factors, bone mineral density (BMD) measurements, and biochemical markers of bone turnover, including serum CTX levels. Early identification of these at-risk individuals is essential to enable timely preventive strategies; in this context, preventive treatment with zoledronic acid may be considered to reduce the risk of skeletal complications and improve overall bone outcomes in selected patients.

In an entirely different clinical context, constitutive activation of canonical Wnt signaling also occurs in a distinct group of non-malignant disorders, notably rare osteosclerotic diseases characterized by excessive bone formation. These conditions, including those caused by pathogenic variants in *SOST* or *LRP5*, are marked by increased bone mass leading to skeletal hypertrophy and, in some cases, compressive neurological complications. Despite sustained Wnt pathway hyperactivation within the skeletal compartment, there is no evidence of an increased incidence of malignancy in affected individuals, suggesting the existence of tissue-restricted regulatory mechanisms that constrain aberrant signaling outside bone.

A similar observation can be made in the therapeutic setting. Although activation of Wnt signaling is a recognized driver in multiple cancers and has prompted the development of pathway inhibitors in oncology, no oncologic safety signal has emerged from clinical trials or post-marketing surveillance of romosozumab. This likely reflects the predominantly bone-specific action of sclerostin inhibition, as sclerostin is largely produced by osteocytes, thereby amplifying Wnt signaling within the bone microenvironment without inducing systemic pathway activation.

Thus, pharmacologic inhibition of Wnt signaling pathway may represent a rational therapeutic strategy in rare osteosclerotic disorders driven by pathological Wnt hyperactivation, where reducing excessive bone formation is a primary therapeutic objective. Supporting this concept, preclinical studies in Wnt-dependent osteosclerotic murine models have demonstrated that treatment with the porcupine inhibitor LGK974 significantly reduces bone mass, highlighting the translational potential of this approach in selected high bone mass diseases [41, 79].

In conclusion, Wnt signaling inhibitors represent a promising class of therapeutics in oncology and fibrotic diseases. However, their use is accompanied by significant skeletal risks, particularly when targeting upstream components such as Wnt ligands or Frizzled receptors. Altogether, optimizing the therapeutic window of Wnt inhibition will require a careful balance between efficacy and skeletal safety, and should be guided by both mechanistic insights and targeted protective strategies.

## Data Availability

This is a literature review, without research data.
